# HumanTestisDB: A Comprehensive Atlas of Testicular Transcriptomes and Cellular Interactions

**DOI:** 10.1093/gpbjnl/qzaf015

**Published:** 2025-03-05

**Authors:** Mengjie Wang, Laihua Li, Qing Cheng, Hao Zhang, Zhaode Liu, Yiqiang Cui, Jiahao Sha, Yan Yuan

**Affiliations:** State Key Laboratory of Reproductive Medicine and Offspring Health, Nanjing Medical University, Nanjing 210029, China; State Key Laboratory of Reproductive Medicine and Offspring Health, Nanjing Medical University, Nanjing 210029, China; Women’s Hospital of Nanjing Medical University, Nanjing Women and Children’s Healthcare Hospital, Nanjing 210000, China; State Key Laboratory of Reproductive Medicine and Offspring Health, Nanjing Medical University, Nanjing 210029, China; State Key Laboratory of Reproductive Medicine and Offspring Health, Nanjing Medical University, Nanjing 210029, China; State Key Laboratory of Reproductive Medicine and Offspring Health, Nanjing Medical University, Nanjing 210029, China; State Key Laboratory of Reproductive Medicine and Offspring Health, Nanjing Medical University, Nanjing 210029, China; Women’s Hospital of Nanjing Medical University, Nanjing Women and Children’s Healthcare Hospital, Nanjing 210000, China; State Key Laboratory of Reproductive Medicine and Offspring Health, Nanjing Medical University, Nanjing 210029, China

**Keywords:** Human testis database, Single-cell RNA sequencing, Cell atlas, Cell–cell interaction, Spermatogenesis

## Abstract

Advances in single-cell technology have enabled the detailed mapping of testicular cell transcriptomes, which is essential for understanding spermatogenesis. However, the fragmented nature of age-specific data from various literature sources has hindered comprehensive analysis. To overcome this, the Human Testis Database (HumanTestisDB) was developed, consolidating multiple human testicular sequencing datasets to address this limitation. Through extensive investigation, 38 unique cell types were identified, providing a detailed perspective on cellular variety. Furthermore, the database systematically categorizes samples into eight developmental stages, offering a structured framework to comprehend the temporal dynamics of testicular development. Each stage features comprehensive maps of cell–cell interactions, elucidating the complex communication network inside the testicular microenvironment at particular developmental stages. Moreover, by facilitating comparisons of interactions among various cell types at different stages, the database enables examining alterations that occur during critical transitions in spermatogenesis. HumanTestisDB, available at https://shalab.njmu.edu.cn/humantestisdb, offers vital insights into testicular transcriptomes and cellular interactions, serving as an essential resource for advancing research in reproductive biology.

## Graphical abstract

**Figure:**
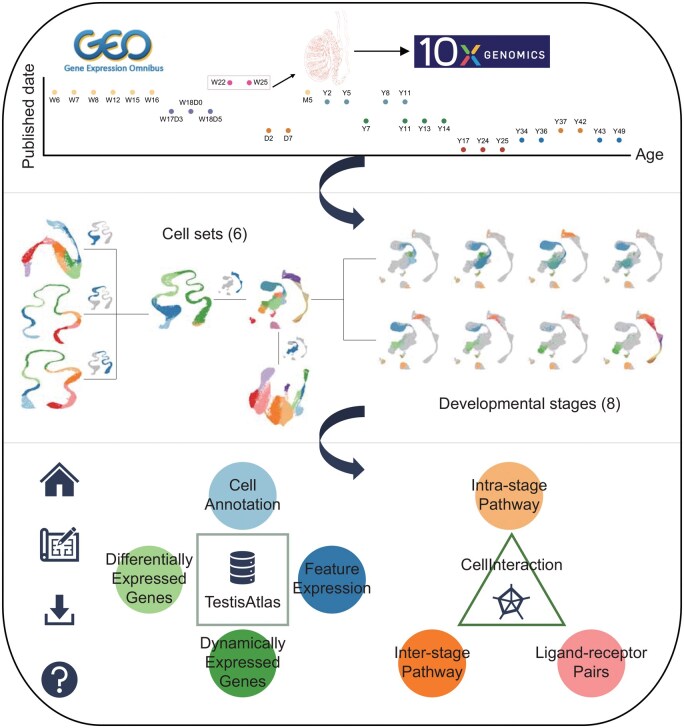

## Introduction

In the male reproductive system, the human testis is essential for both sperm production and hormone regulation. It includes various cell types, all of which are essential to the spermatogenesis process [1–3]. Single-cell technologies have advanced significantly in recent years, offering previously unheard-of insights into the testicular microenvironment’s functional dynamics and cellular heterogeneity [4–11]. These technologies have been applied to human fetal germ cells (FGCs), demonstrating that the development of male FGCs undergoes stages including migration, mitosis, and cell-cycle arrest [4]. The transcriptome profiles of adult testes were then generated, describing the different states of spermatogonia and analyzing gene expression changes throughout male meiosis and spermiogenesis [5–7]. Following the construction of the transcriptome maps of the testes during embryonic, fetal, childhood, and adolescent stages, further understanding of the development of somatic cells, particularly Sertoli and Leydig cells, was achieved [8–11]. Despite these developments, the testicular sequencing data remain unintegrated and fail to illustrate the distribution of cell types across various ages. Furthermore, alternative analytical methodologies employed in diverse literature yield inconsistent results.

This study tackles the significant deficiency in consolidating and analyzing disparate testicular cell data. Our contribution, HumanTestisDB, signifies a substantial advancement in the compilation and augmentation of existing testicular cell datasets with comprehensive annotations. The purpose of this database is to serve as a repository and to offer a framework for comprehending the complex cell–cell interactions within the testicular microenvironment at different developmental stages. HumanTestisDB provides essential resources for researchers and clinicians to investigate the intricate cellular processes and molecular pathways underlying testicular development and function. Infertility impacts a large number of couples globally, with male factor infertility accounting for a substantial share [12,13]. However, the primary reasons for male infertility, including azoospermia (the lack of sperm in semen), remain inadequately understood [14]. Researchers can better understand the molecular basis of male infertility by comparing the HumanTestisDB data with sequencing data from azoospermia patients. This understanding could inform future research, encourage individualized medical approaches to infertility treatment, and eventually improve the reproductive outcomes of those affected.

In this study, multiple human testicular sequencing datasets covering a broad age range were methodically combined to develop HumanTestisDB. It consists of 38 different cell types, providing a thorough understanding of the cellular composition of the testis. After classifying samples from different ages into eight stages, the complex cellular composition and interactions within each stage were analyzed. The molecular mechanisms underlying testicular development and function can be better understood using this stratification to uncover universal and stage-specific signaling pathways.

## Database construction

### Data collection and preprocessing

Among the 46 datasets included in HumanTestisDB, three datasets (W22, W25A, and W25B) were generated by our laboratory by performing 10X Genomics sequencing on donated testicular samples, while the other 43 datasets were sourced from published studies (Table S1). The literature-based data were identified using keywords such as “human testis”, “scRNAseq”, and “10X Genomics”. From these studies, we obtained Sequence Read Archive (SRA) files, converted them to FASTQ format using the fastq-dump command from SRAtoolkit (v2.10.8, https://github.com/ncbi/sra-tools), and processed them with CellRanger (v5.0.0) [15] to generate feature-barcode matrices. When FASTQ files were unavailable, we directly acquired feature-barcode matrices from the publications. The laboratory-generated data were similarly processed with CellRanger (v5.0.0).

### Human fetal gonad preparation for single-cell RNA sequencing

Male fetal gonads were washed three times with phosphate buffered saline (PBS) and divided into smaller sizes (around 2 mm each) using scissors. Single testicular cells were obtained using two-step enzymatic digestion. Briefly, tissues were treated with collagenase type IV (Catalog No. 17104, GIBCO, Waltham, CA) for 10–20 min followed by 0.25% EDTA (Catalog No. 25200056, GIBCO) for 3–8 min at 37°C. Then, the digestion was stopped by adding 10% fetal bovine serum (FBS; Catalog No. 10082147, GIBCO). Single testicular cells were obtained by filtering through a 70-μm strainer (Catalog No. 08-771-2, Thermo Fisher Scientific, Waltham, MA) and a 40-μm strainer (Catalog No. 08-771-1, Thermo Fisher Scientific). The cells were pelleted by centrifugation at 600–800 *g* for 5 min and washed with PBS twice. Cell number was counted using cell counter (Catalog No.C100-SE, RWD, Shenzhen, China), and the cells were then resuspended in PBS supplemented with 0.4% bovine serum albumin (BSA; Catalog No. AM2616, Thermo Fisher Scientific) at a concentration of ∼ 1000 cells/µl, ready for single-cell RNA sequencing (scRNA-seq).

### Library preparation and scRNA-seq

We aimed to capture ∼ 4000–5000 cells. Briefly, cells were diluted following manufacturer’s instructions, and 70 µl of total mixed buffer together with cells were loaded into 10X Chromium Controller (Catalog No. GCG-SR-1, 10X Genomics, Pleasanton, CA) using the Chromium Single Cell 3′ v3.1 reagents (Catalog No. PN-1000121, 10X Genomics). In brief, single-cell suspensions were used for gel bead in emulsion (GEM) generation, GEM-RT, and cDNA amplification and purification. cDNA concentrations were measured with Qubit dsDNA HS and BR Assay Kits (Catalog No. Q32584, Thermo Fisher Scientific). Indexed libraries were constructed according to the user guide. Post-library construction quality control was performed on a Fragment Analyzer or LabChip, and the library products were sequenced on an Illumina NovaSeq 6000 sequencer at the Nanjing Jiangbei New Area Biopharmaceutical Public Service Platform (Nanjing, China) with the following sequencing strategy: 150-bp read length for paired-end.

### Data quality control

Each feature-barcode matrix was converted into a Seurat object using the CreateSeuratObject function from the SeuratObject package (https://github.com/satijalab/seurat-object). Quality control was conducted using the RunCellQC function of the SCP package (https://github.com/zhanghao-njmu/SCP). We applied scDblFinder [16] for doublet detection, as it is common to have 10%–20% doublets in single-cell experiments. Low-quality cells were filtered out based on median absolute deviation (MAD) [17]. The retained cells met the following standards: percent.mito < 20, percent.ribo < 50, nFeature_RNA > 500, and nCount_RNA > 1000.

### Batch effect correction

To mitigate batch effects from different sources, we first reproduced the dimensionality reduction results of each literature to determine the magnitude of batch effects among the datasets. We divided 46 datasets into 14 groups, namely: Group1 (W6, W7, W8, W12, W15, and W16), Group2 (W17D3, W18D0, and W18D5), Group3 (W22, W25A, and W25B), Group4 (D2-I, D2-T, D7-I, and D7-T), Group5 (M5), Group6 (Y7-rep1 and Y7-rep2), Group7 (Y2, Y5, and Y8), Group8 (Y17-1-rep1, Y17-1-rep2, Y24-rep1, Y24-rep2, Y25-rep1, and Y25-rep2), Group9 (Y11-1-rep1, Y11-1-rep2, Y13-rep1, Y13-rep2, Y14-rep1, and Y14-rep2), Group10 (Y11-2), Group11 (Y43-2-Spc and Y43-2-Spd), Group12 (Y34-1, Y36-1, and Y49-1), Group13 (Y43-1-Spc and Y43-1-Spd), and Group14 (Y37-2-I, Y37-2-T, Y42-1-I, and Y42-1-T). We passed the grouping information to the “batch” parameter of the Integration_SCP function in the SCP package and set nHVF = 870 and integration_method = “CSS” [18] for data integration.

### Cell type identification

Cell types were deduced from the integrated data based on a combination of criteria: classical cell type-specific markers (*e.g.*, *AMH* for Sertoli cells and *DDX4* for germ cells), annotations from publications (*e.g.*, Figure S4D), and additional factors (*e.g.*, cell cycle phase and gene content). Gene Ontology (GO) [19] enrichment analysis also contributed to this identification process.

### Differentially expressed gene identification

Differentially expressed genes of each cell type were identified using the RunDEtest function of the SCP package with default parameters (test.use = “wilcox”, fc.threshold = 1.5, base = 2, min.pct = 0.1, p.adjust.method = “Bonferroni”), considering all genes with adjusted *P* value < 0.05 as differentially expressed.

### Dynamically expressed gene identification

First, the RunSlingshot function of the SCP package was used to compute the pseudotime of cells. The parameters “reduction”, “group.by”, and “start” were used to specify the nonlinear dimensionality reduction graph, cell group, and the cell group to serve as the starting point, respectively. Second, the calculated pseudotime was passed to the “lineages” parameter of the RunDynamicFeatures function in the SCP package, with n_candidates set to 5000 to identify dynamically expressed genes.

### Gene set enrichment analysis and gene annotation

The RunEnrichment function of the SCP package was used for gene set enrichment analysis, with GO terms and Reactome pathways as background gene sets [db = c (“GO_BP”, “Reactome”), minGSSize = 10, maxGSSize = 500, GO_simplify = TRUE, GO_simplify_padjustCutoff = 0.2, simplify_method = “Rel”, simplify_similarityCutoff = 0.7]. The transcription factors, cell surface proteins, and enzymes used for gene annotation were obtained through the PrepareDB function of the SCP package (species = “Homo_sapiens”, db = “TF” or “SP” or “Enzyme”) and are available in the “Download” module of HumanTestisDB.

### Cell cycle-related analysis and cell relationship inference

Cell cycle phases were determined using the CellCycleScoring function of the Seurat [20] package. The genes related to S/G2M phase were obtained using the CC_GenePrefetch function of the SCP package. The cell-cycle-arrest-related genes were obtained from Li and colleagues [4]. These cell-cycle-related genes are also available in the “Download” module of HumanTestisDB. The RunPAGA function of the SCP package was used to infer the strength of the correlations between cells, with parameters “group_by”, “linear_reduction”, and “nonlinear_reduction” used to specify cell groups, linear dimensionality reduction graphs, and nonlinear dimensionality reduction graphs, respectively.

### Cell communication

Before analyzing cell–cell interactions, we refined cell type naming for accuracy. Criteria for retaining cell types included: (1) a proportion greater than 1.3% for continuously differentiating cells and (2) a cell count above 10 for terminally differentiated cells. Red blood cells and epithelial cells were excluded. For each developmental stage, cell–cell communication was then analyzed using the CellChat [21] package following the official tutorial (https://htmlpreview.github.io/?https://github.com/jinworks/CellChat/blob/master/tutorial/CellChat-vignette.html), with all identified interactions retained. When comparing cell communication across stages, we extracted the results of each stage and merged them into a matrix. Based on the analysis purpose, we divided each value in the matrix by the row or column sums to obtain relative communication strength. In addition, the terms “Incoming signaling patterns” and “Outgoing signaling patterns” involved in the pathway analysis were defined by the CellChat package, as stated by Jin et al. [22]: “Outgoing patterns reveal how sender cells (that is, cells acting as signal sources) coordinate with each other, as well as how they coordinate with certain signaling pathways to drive communication. Incoming patterns show how target cells (that is, cells acting as signal receivers) coordinate with each other to respond to incoming signals.”

### Database implementation

HumanTestisDB was developed in a container based on docker’s r-base image [23]. The container’s environment was configured using apt and pip. In this loosely isolated environment, website was constructed with Django (https://www.djangoproject.com/), in which data analysis was performed using R programming language, while backend to frontend interaction was managed using Python and HTML. Accessible at https://shalab.njmu.edu.cn/humantestisdb, the database provides a user-friendly interface for exploring cell type distribution, gene expression patterns, and cell–cell interactions.

## Database content

### Overview of HumanTestisDB

**Figure 1:**
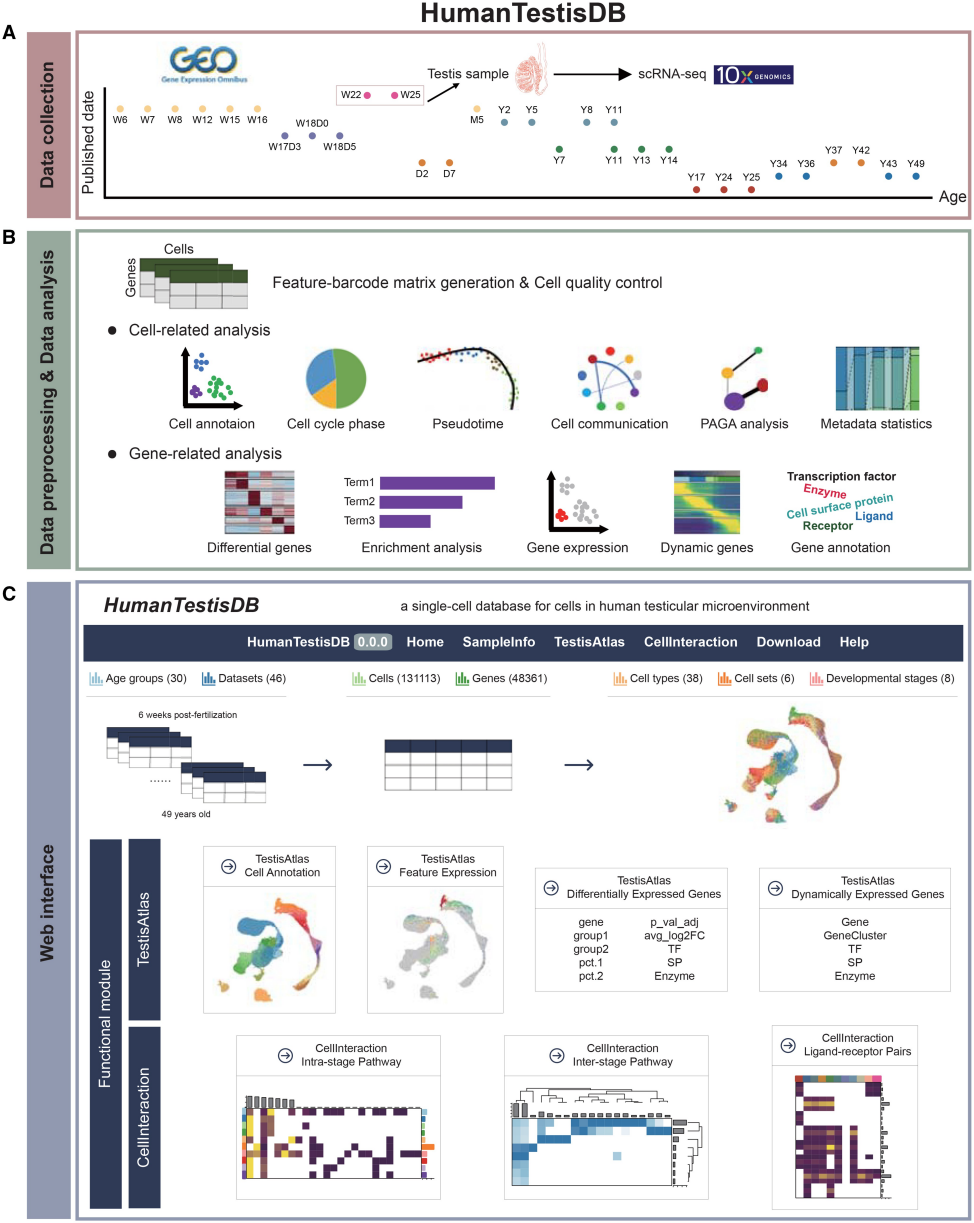
**Schematic diagram of the overall design of HumanTestisDB** **A**. The sources and sample ages of collected data. **B**. Bioinformatics analysis methods used for processing collected data. **C**. The user interface. W, weeks post-fertilization; D, days postnatal; M, months postnatal; Y, years postnatal; scRNA-seq, single-cell RNA sequencing; PAGA, partition-based graph abstraction.

**Figure 2:**
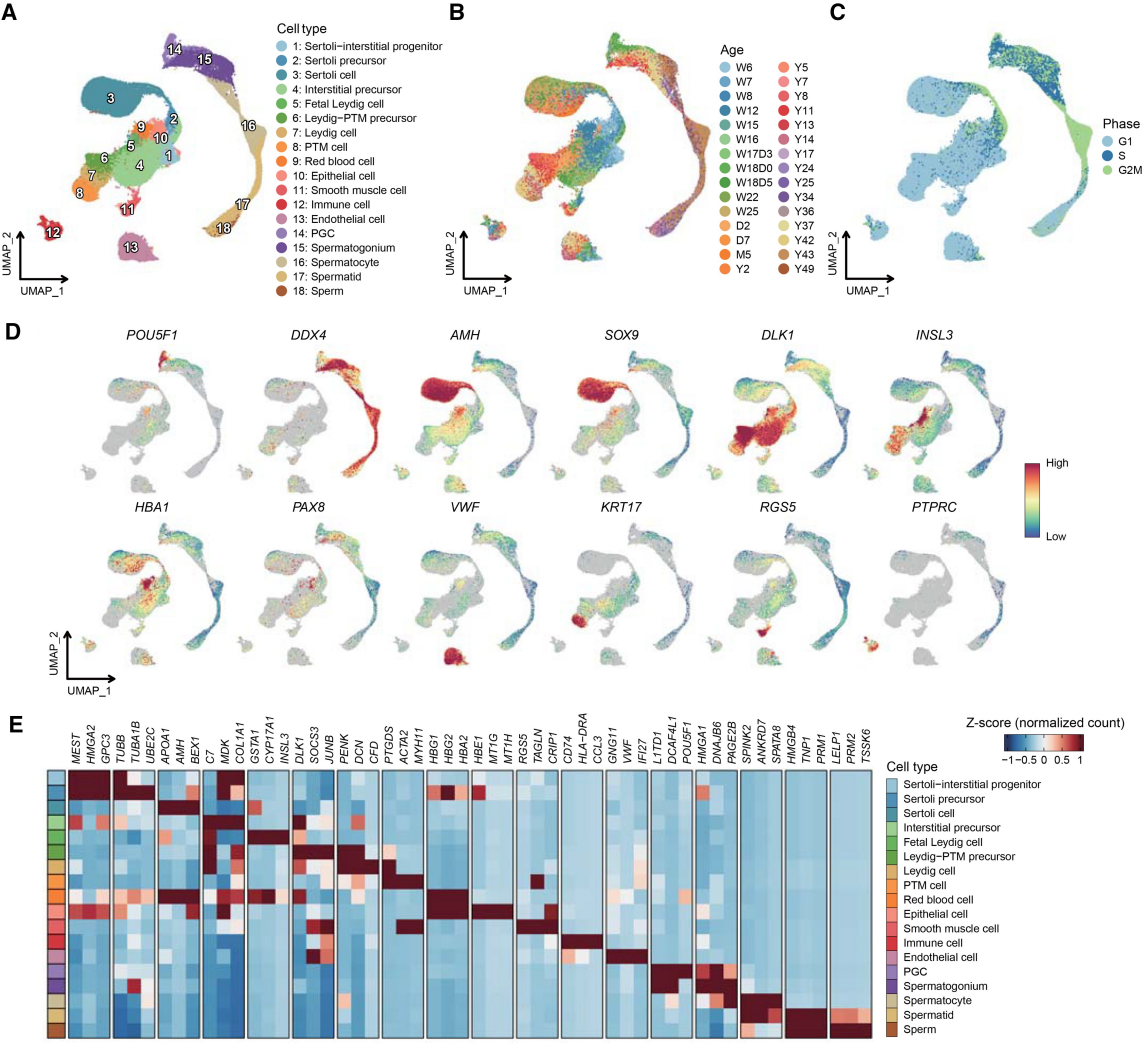
Global transcriptome profiling of all testicular cells in HumanTestisDB **A**.–**C**. UMAP plots of integrated data from W6 to Y49. Each point represents a single cell and is colored according to cell type (A) or age (B) or cell cycle phase (C). **D**. Expression patterns of classical marker genes for major cell types in (A). **E**. Heatmap of expression levels for top 3 (according to fold change) differentially expressed genes of each cell type in (A). Each row represents a cell type, and each column represents a gene. UMAP, uniform manifold approximation and projection; PTM, peritubular myoid; PGC, primordial germ cell.

HumanTestisDB is a comprehensive database that includes 46 scRNA-seq datasets from human testes, ranging from 6 weeks post-fertilization (W6) to 49 years of age (Y49) (**Figure 1**A and **Figure 2**B; Table S1). Following quality control, our final dataset comprised 131,113 cells (Figure S1). Employing established marker expression patterns (Figure 2D and E, Figure S2), these cells were initially classified into 18 distinct cell types (Figure 2A). This classification comprised 13 somatic and 5 germ cell types (Figure 2A).

GO enrichment analysis was conducted to confirm our cell type classification, concentrating on the differentially and highly expressed genes in each cell type (Figure S3). The results showed a notable enrichment of GO terms that closely matched each cell type’s inherent properties. Specifically, spermatocytes and spermatids showed significant enrichment in processes associated with meiosis and spermatid differentiation, respectively. Furthermore, Sertoli precursors demonstrated a substantial correlation with chromosome segregation-related terms, consistent with our finding that most of these cells are in the S/G2M phase of the cell cycle (Figure 2C).

**Figure 3:**
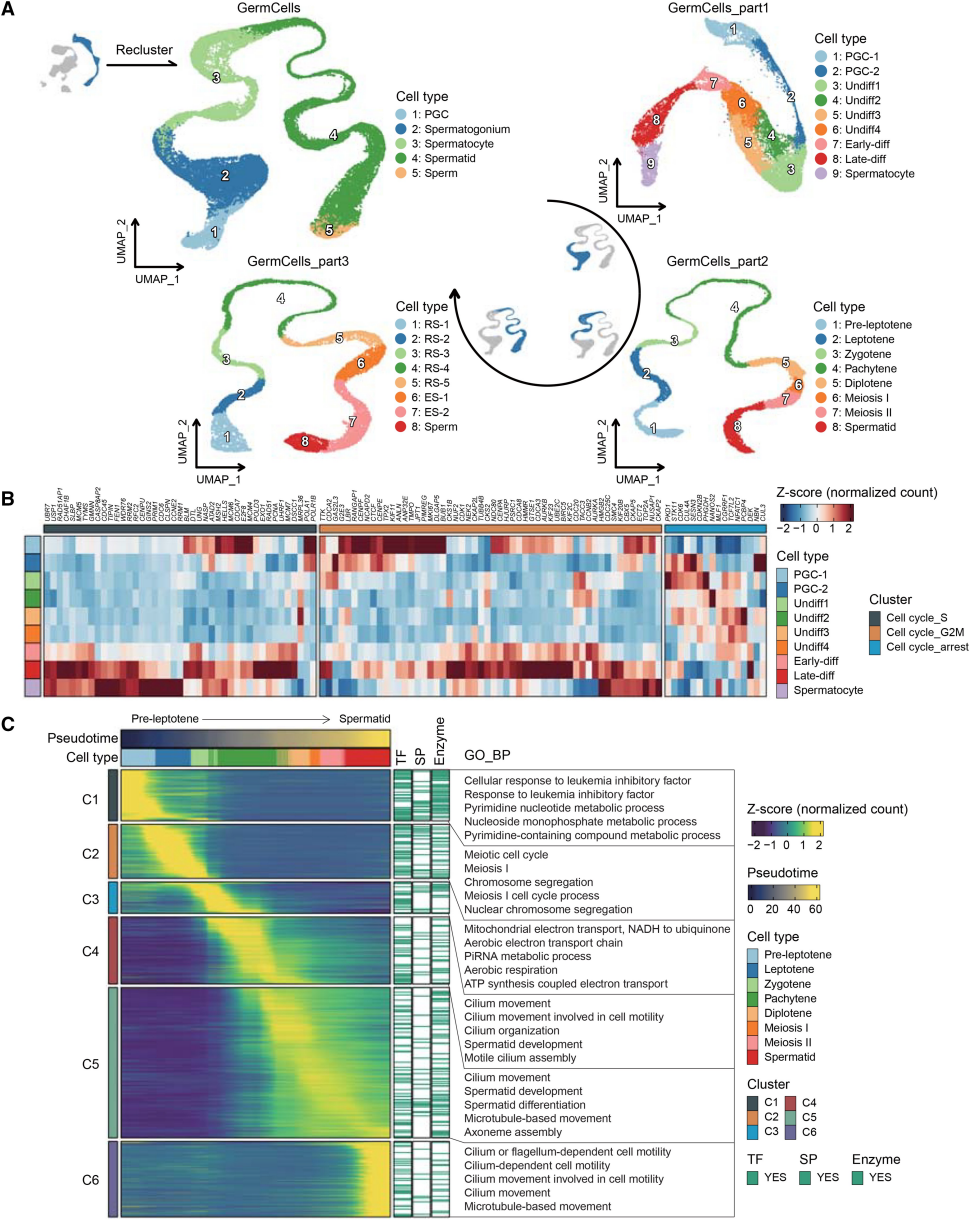
**Case study of human testicular germ cells (from PGCs to immature sperm)** **A**. UMAP plots of germ cells at various spermatogenesis stages, colored by cell type. **B**. The expression levels of cell-cycle-related genes during the transition from PGC-1 to early spermatocytes. **C**. Heatmap showing genes dynamically expressed along the developmental trajectory from pre-leptotene to spermatid. Genes categorized as TFs, cell SPs, or enzymes are highlighted in green; associated GO BP terms are given on the right of the corresponding gene clusters. Undiff, undifferentiated spermatogonium; Early-diff/Late-diff, early/late differentiated spermatogonium; RS, round spermatid; ES, elongated spermatid; GO, Gene Ontology; TF, transcription factor; SP, surface protein; BP, biological process.

**Figure 4:**
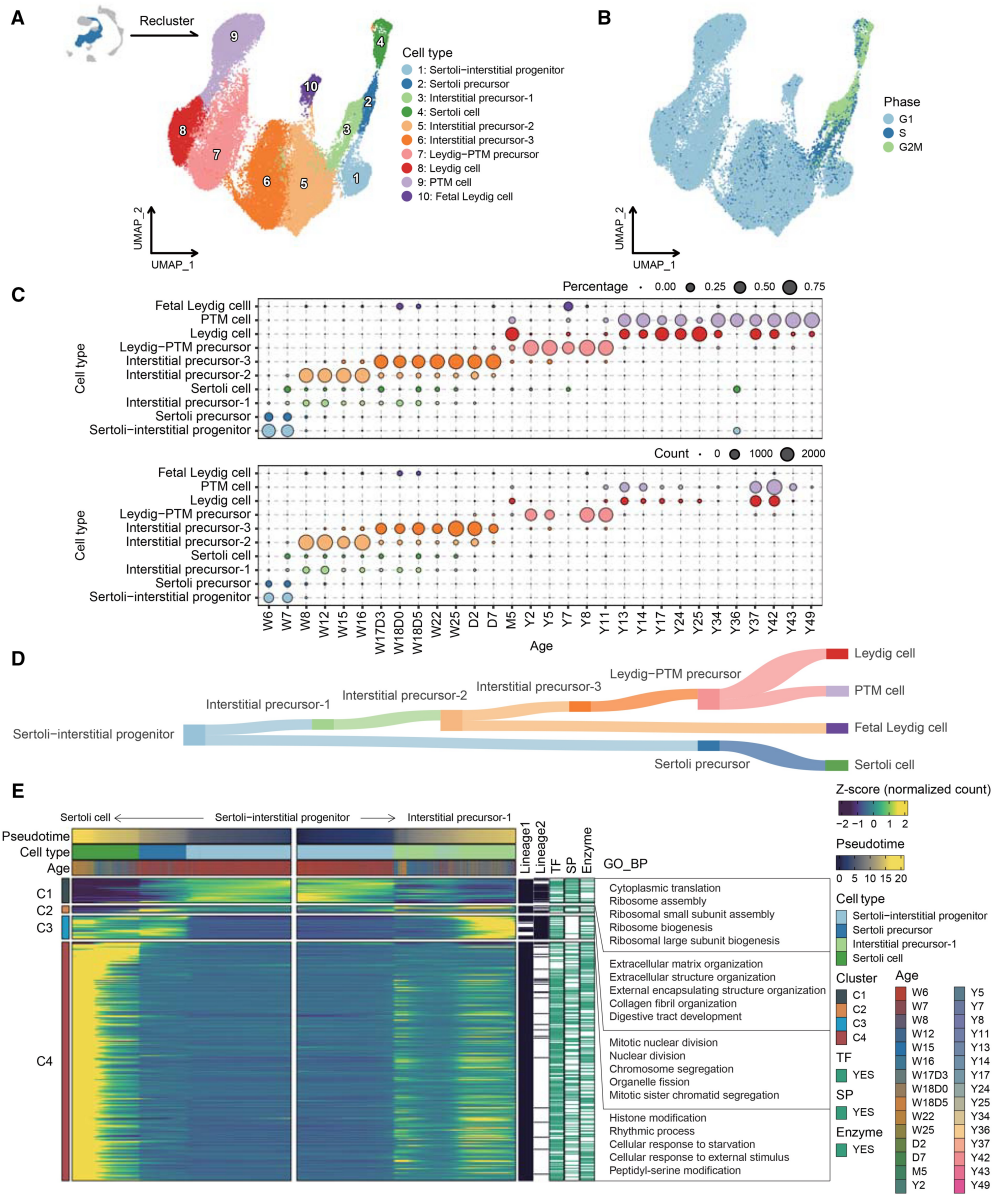
**Case study focusing on human testicular somatic cells** **A**. and **B**. UMAP plots obtained by reclustering interstitial-associated cells and early Sertoli cells in Figure 2A, colored by cell type (A) or cell cycle phase (B). **C**. Statistical plot showing the distribution of each somatic cell type in each age group. The size of the dots is proportional to the percentage of cells (upper row) or the number of cells (lower row). **D**. Schematic illustration of somatic cell development trajectory. **E**. Heatmap illustrating dynamically expressed genes along developmental trajectories (from Sertoli–interstitial progenitor to Sertoli cell and from Sertoli–interstitial progenitor to interstitial precursor-1), with TFs, cell SPs, or enzymes highlighted in green and enriched GO BP terms for each gene cluster displayed.

**Figure 5:**
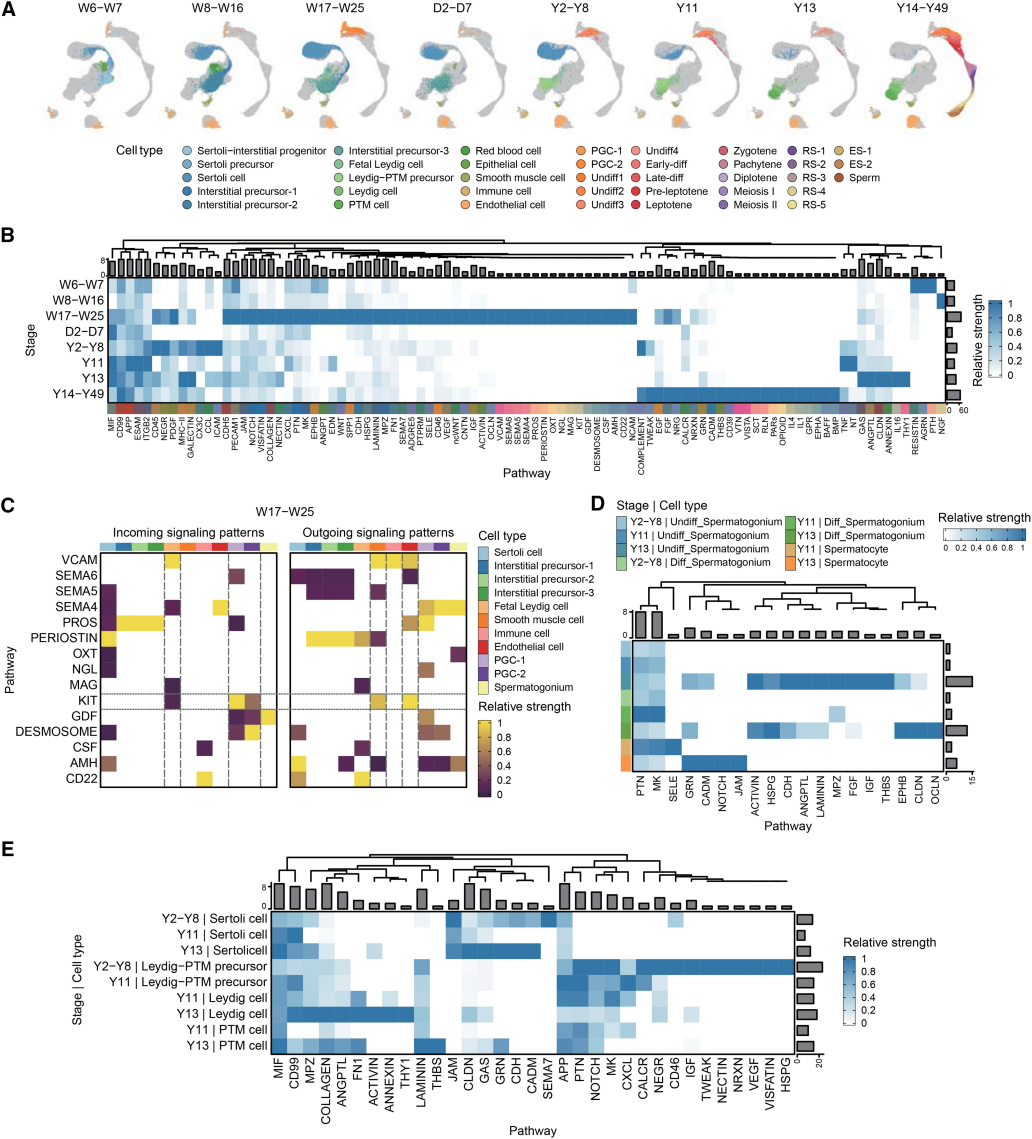
**Cell–cell communication dynamics within the testicular microenvironment** **A**. UMAP plots of eight distinct developmental stages that differ in cell type composition, colored by cell type. **B**. Heatmap depicting relative strength of signaling pathways common or specific to stages, with normalized columns for comparability. Each row represents a stage, and each column represents a pathway. The height of the bars at the top represents the number of stages in which each pathway is detected. The width of the bars on the right represents the number of pathways detected within each stage. **C**. Heatmap illustrating receivers and senders mediating cell–cell communication specific to stage W17–W25, with normalized columns for signal strength comparability. Each row represents a pathway, and each column represents a cell type. **D**. Heatmap presenting communication strength of signaling pathways received by different germ cell types at specified stages, with normalized columns for comparability. Each row represents a cell type, and each column represents a pathway. The height of the bars at the top represents the number of cell types that receive each pathway. The width of the bars on the right represents the number of pathways received by each cell type. **E**. Heatmap showcasing communication strength of signaling pathways sent by different somatic cell types at specified stages, with normalized columns for comparability. Each row represents a cell type, and each column represents a pathway. The height of the bars at the top represents the number of cell types that send each pathway. The width of the bars on the right represents the number of pathways sent by each cell type.

The collection of all 131,113 cells in the database is designated as “AllCells” (Figure 2A). In addition to this large cell set, cells associated with key biological events of spermatogenesis were isolated from the “AllCells” set, forming several subsets named “GermCells”, “GermCells_part1”, “GermCells_part2”, “GermCells_part3”, and “SomaticCells”. “GermCells” comprises all germ cells; “GermCells_part1” includes primordial germ cells (PGCs), spermatogonia, and early spermatocytes; “GermCells_part2” consists of spermatocytes and early spermatids; “GermCells_part3” encompasses spermatids and immature sperm (**Figure 3**A); and “SomaticCells” refers to cells derived from the Sertoli–interstitial progenitor lineage (**Figure 4**A). Several analyses were performed on these cell sets (Figure 1B), and the findings were employed to categorize the 46 samples in the database, from W6 to Y49, into eight developmental stages (**Figure 5**A).

We provide our findings via an accessible website comprising six modules: “Home”, “SampleInfo”, “TestisAtlas”, “CellInteraction”, “Download”, and “Help”. The primary functional modules are “TestisAtlas” and “CellInteraction”. The “TestisAtlas” module facilitates comprehensive inquiries into each cell set, encompassing cell attributes, gene expression profiles, differentially expressed genes for each cell type, and genes expressed dynamically throughout cell development. The “CellInteraction” module describes the signaling pathways, ligand–receptor pairs, and cell types involved in the intercellular interactions in the testicular microenvironment at various developmental stages (Figure 1C).

In summary, HumanTestisDB is a database presented through a user-friendly website that contains the results of multiple analyses of single-cell transcriptomes of the human testis.

## Database application

### Proliferation and differentiation of germ cells

HumanTestisDB is primarily utilized to investigate transcriptomic changes throughout spermatogenesis. This database contains four subsets of germ cells: “GermCells”, “GermCells_part1”, “GermCells_part2”, and “GermCells_part3”. “GermCells” delineates the progression from PGC to immature sperm, while “GermCells_part1”, “GermCells_part2”, and “GermCells_part3” outline the three essential stages of spermatogenesis: PGC differentiation and spermatogonium mitosis, spermatocyte meiosis, and spermiogenesis, respectively (Figure 3, Figures S4–S6). The “TestisAtlas” module comprises four functional pages: “Cell Annotation”, “Feature Expression”, “Differentially Expressed Genes”, and “Dynamically Expressed Genes” (Figure 1C), allowing users to select the preferred cell set through the “Cell Set” option.

“GermCells_part1” comprises two types of PGCs, six types of spermatogonia, and early spermatocytes (Figure 3A, Figure S4A and B). PGC-1 cells exhibited the expression of pluripotency markers *POU5F1* and *NANOG*, whereas PGC-2 cells lacked *POU5F1* expression but expressed *DDX4* (Figure S4C), aligning with findings documented in prior research about FGCs [24–28]. Analysis of undifferentiated spermatogonium (Undiff) showed that Undiff2 mainly expressed genes related to cytoplasmic translation and ATP synthesis. In contrast, Undiff3 was involved in various biological processes, such as cell cycle progression [29], signal transduction [30,31], inflammatory responses [32–34], and spermatogonium migration induction [35] (Figure S4E; Table S2). After that, undifferentiated spermatogonia progressed to the differentiation stage and initiates meiosis (Figure S4B). A distinct trend was identified when examining the expression of cell-cycle-related genes in PGC-1 cells to early spermatocytes (Figure 3B). PGC-1 cells expressed genes associated with the S and G2M phases, whereas PGC-2 to Undiff4 cells exhibited increased expression of genes related to cell-cycle arrest compared to PGC-1 cells. In early differentiated spermatogonia to early spermatocytes, the expression of genes associated with cell-cycle arrest diminished. However, the expression of genes pertinent to the S and G2M phases significantly increased (Figure 3B). This aligns with the research indicating that early PGCs are in an active proliferation phase, late PGCs undergo mitotic suppression, and male germ cells do not initiate meiosis until adolescence [4,36,37].

HumanTestisDB provides comprehensive information on spermatocyte stages in “GermCells_part2” that spans pre-leptotene to meiosis II, which differs from many other studies (Figure 3A). The initiation of meiosis is characterized by a majority of cells in the S phase and increased expression of mitochondrial genes (Figure S5A and C). Furthermore, HumanTestisDB enables users to observe significant changes in gene expression patterns throughout these stages, with specific gene clusters associated with various biological processes (Figure 3C). The C1 gene set is mainly expressed in pre-leptotene and participates in the pyrimidine nucleotide metabolic process, which is consistent with the fact that a large amount of DNA is synthesized in pre-leptotene. The C2 gene set is mainly expressed in leptotene and zygotene, and these genes are enriched in terms related to meiosis. The C3 gene set, primarily expressed in zygotene and pachytene, shows strong involvement in ATP synthesis and piRNA metabolism. Kawase et al. showed that testicular piRNA can be divided into fetal piRNA, pre-pachytene piRNA, and pachytene piRNA [38], indicating the reliability of our enrichment results. The C4 gene set is predominantly expressed during pachytene, a stage characterized by the close association of homologous chromosomes and the exchange and recombination of incomplete DNA segments between alleles. The GO enrichment results demonstrate that the C4 gene set is associated with cilia. Xie et al. showed that deleting germ cell-specific ciliary genes could increase germ cell apoptosis, reduce crossover formation, and damage double-strand break repair [39]. The C5 gene set is expressed from diplotene to early spermatids, exhibiting the most prominent expression peak across several cell types. These genes are implicated in spermatid development and are enriched in terms associated with sperm structure. The C6 gene set is expressed explicitly in early spermatids, with enrichment for terms associated with sperm structure (Figure 3C).

As observed in “GermCells_part3” (Figure 3A), the last stage, spermiogenesis, entails turning spermatids into immature sperm. According to HumanTestisDB, chromatin condensation and subsequent transcriptional shutdown lead to a decrease in gene expression as round spermatids give way to elongated spermatids (Figure S6B), a phenomenon also observed in mice [40]. The “Dynamically Expressed Genes” page in the “TestisAtlas” module lists genes that exhibit dynamic expression throughout the transition from round spermatids to immature sperm, grouping them into six clusters based on their expression patterns (Figure S6C). Following their departure from meiosis and subsequent spermatid transformation, early spermatids exhibit differential expression of genes associated with cell division and spermatid development (Figures S5B, S6A, and S6C). Using the gene data from GeneCards, the relationship between each of the 1311 genes in the last five clusters and spermiogenesis-related events was deduced, providing crucial new information for future spermatid biogenesis and function research (Figure S6C; Table S3).

The four germ cell subsets in HumanTestisDB enable comprehensive analysis of the complete process or specific aspects of spermatogenesis, which is crucial for elucidating the molecular mechanisms of sperm production and discovering key regulatory genes.

### Key roles of Sertoli and Leydig cells in the seminiferous tubule microenvironment and their developmental trajectories

Sertoli and Leydig cells play crucial roles in forming and maintaining the seminiferous tubule microenvironment [41–45]. HumanTestisDB, a critical tool for advancing testicular research, enables the study of germ cells and enhances understanding of somatic cell differentiation. The “SomaticCells” cell set was created to investigate transcriptomic changes during the differentiation of these somatic cells (Figure 4A). During the critical period of 6 to 7 weeks post-fertilization, the predominant cell type identified was the Sertoli–interstitial progenitor (Figure 4C). These progenitors subsequently differentiate into Sertoli or interstitial cells (Figure 4A). Following the commitment to the interstitial cell lineage, an intriguing transition occurs, with cells differentiating into fetal Leydig, mature Leydig, and peritubular myoid (PTM) cells. The differentiation process is characterized by unique gene expression patterns, which are accessible in the “TestisAtlas” module (Figure 4D and E, Figure S7).

Investigating the “Cell Annotation” page under the “TestisAtlas” module yielded additional insights (Figure 1C). Among these developing cell types, a distinct age-related distribution pattern was observed (Figure 4C). In particular, stages W8–W16 and W17D3–D7 represent the periods when interstitial precursor-2 and interstitial precursor-3 are most prevalent. Interestingly, the predominant cell type before puberty is Leydig–PTM precursors. After puberty, these precursors differentiate into Leydig and PTM cells, undergoing a significant shift. Furthermore, most Sertoli precursors, interstitial precursor-1 cells, and early Sertoli cells were found to be in the proliferation phase, specifically the S and G2M phases. On the other hand, other cell types mainly reside the G1 phase of the cell cycle (Figure 4B).

In conclusion, the “SomaticCells” cell subset offers a comprehensive illustration of the Sertoli–interstitial progenitor’s developmental trajectory, enhancing our understanding of somatic cell differentiation and providing the foundation for future research into the characteristics of somatic cells at various stages.

### Intricate cell–cell communication in the testicular microenvironment across developmental stages

The testis is a complex organ with diverse cell types and a unique physical structure. Since most testicular cell types are functionally interdependent, normal spermatogenesis depends on their interactions [46–48]. HumanTestisDB describes the developmental process from W6 to Y49, during which testicular cell types undergo significant changes, particularly in germ and interstitial-derived cells. Based on the distribution of various cell types in these samples, W6–Y49 may be categorized into eight developmental stages. (1) W6–W7: the germ cells are all PGC-1 cells, while the interstitial-derived cells consist primarily of Sertoli–interstitial progenitors with a small number of interstitial precursors. (2) W8–W16: most of the interstitial-derived cells are interstitial precursors, with interstitial precursor-2 cells being predominant. A tiny number of PGC-2 cells are present compared to stage W6–W7. (3) W17D3–W25: compared to stage W8–W16, there are significantly more PGC-2 cells and a few spermatogonia. Most interstitial-derived cells are interstitial precursors, predominantly interstitial precursor-3. (4) D2–D7: compared to stage W17D3–W25, there are significant changes in the distribution of endothelial and smooth muscle cells. (5) Y2–Y8: PGCs are absent, and most germ cells are undifferentiated spermatogonia with limited differentiated spermatogonia. Most interstitial-derived cells are Leydig–PTM precursors. (6) Y11: there is an increase in the number of differentiated spermatogonia compared to stage Y2–Y8, along with the emergence of limited spermatocytes. Most interstitial-derived cells are Leydig–PTM precursors, with limited Leydig and PTM cells. (7) Y13: compared to stage Y11, limited spermatids appear, whereas interstitial-derived cells consist of Leydig and PTM cells. (8) Y14–Y49: in contrast to stage Y13, there exists a diverse array of germ cells (from spermatogonia to immature sperm), while interstitial-derived cells include Leydig and PTM cells (Figure 5A, Figures S8 and S9).

This study investigated cell–cell interactions at each developmental stage utilizing the CellChat software. Specific signaling pathways, including CD99, APP, and ESCM, demonstrate sustained activity across all eight stages (Figure 5B). We also uncovered stage-specific pathways. For example, 15 different pathways, including VCAM and CD22, are primarily active during stage W17–W25 (Figure 5B).

The “Intra-stage Pathway” page in the “CellInteraction” module comprehensively outlines the signaling pathways identified at each developmental stage and the associated sender and receiver cells. Compared to the “Intra-stage Pathway” page, the “Ligand-receptor Pairs” page explores more deeply, providing detailed insights into the ligand–receptor pairs (Figure 1C). Focusing on stage W17–W25, HumanTestisDB validates prior studies, emphasizing that PGCs express the *KIT* gene (encoding a transmembrane tyrosine kinase receptor) [49–54]. Furthermore, it demonstrates that *KITL*, encoding the ligand for KIT, is mainly expressed by endothelial and smooth muscle cells, and fetal Leydig cells are the main recipients of KIT signaling (Figure 5C). This observation confirms the essential function of KITL-KIT signaling in development and maintenance of fetal Leydig cells [55].

Unlike the “Intra-stage Pathway” and “Ligand-receptor Pairs” pages, the “Inter-stage Pathway” page allows for cross-stage comparisons of signals received or sent by cell types (Figure 1C). For example, during meiosis, a process that begins at the commencement of puberty in males and is critical for producing the large number of gametes required for male fertility, HumanTestisDB reveals a different development of germ cells into spermatocytes at stages Y11 and Y13, as opposed to stage Y2–Y8. Specifically, stage Y11, unlike stage Y13, continues to include Leydig–PTM precursors (Figure 5A, Figures S8 and S9). The present study compared the signals received by germ cells and those sent by the primary somatic cells during these three stages (Figure 5D and E). Analysis of signal strength suggests that spermatogonia at stage Y13 receive a more comprehensive range of signals than their earlier-stage counterparts, including ACTIVIN, IGF, and EPHB (Figure 5D). Notably, research reveals that *IGF1* plays an essential role in controlling spermatogenesis and encouraging the differentiation of spermatogonia into primary spermatocytes [56–58].

In conclusion, HumanTestisDB provides a verifiable repository of signaling pathways, thereby facilitating advanced *in vitro* germ cell culture research.

## Discussion

HumanTestisDB is a comprehensive database, though its scope is constrained by the limited variety of datasets available. A main limitation is the underrepresentation of Sertoli cells in samples from puberty to adulthood. According to Guo et al., mature Sertoli cells generally exceed the size range effectively recorded by 10X Genomics technology [5], leading to an inadequately low representation of adult Sertoli cells in the database. This highlights the necessity of incorporating more comprehensive Sertoli cell sequencing data, which would significantly enhance the content and functionality of HumanTestisDB.

Future database upgrades should incorporate a broader range of demographic data to enhance its relevance and applicability. As single-cell technologies advance, it is essential to keep the database updated by including new findings and methodological innovations. These upgrades would improve the database’s research applicability and enable better translation of these findings into clinical settings, especially for male reproductive health and the management of associated disorders.

Furthermore, investigation of the epigenetic variables influencing testicular development is a promising but unexplored field. Exploring this aspect of testicular transcriptomics is a promising avenue for future research, with the potential to identify new treatment targets and mechanisms in male reproductive health.

## Conclusion

An important step forward in understanding the complexities of the human testicular microenvironment is represented by HumanTestisDB. The fact that it was created highlights the growing need for integrative databases to summarize and interpret biological systems’ complex nature. By combining disparate data, HumanTestisDB goes beyond being a simple archive and improves our understanding of testicular biology. It provides a comprehensive transcriptome atlas of the human testis, tracing its development from embryonic stage to adulthood, by integrating 46 scRNA-seq datasets.

The careful identification of 38 different cell types and the explanation of their developmental trajectories constitute the foundation of HumanTestisDB’s originality. This resource is revolutionary because it provides previously unheard-of insights into the cellular mosaic of human tissues. Furthermore, the database plays a crucial role in deciphering the complex communication network that permeates different testicular cells at various developmental stages. The focus on developmental stratification is particularly significant, reflecting the changing viewpoint in developmental biology that recognizes cell states’ fluid and dynamic characteristics. Its contributions are essential in promoting further research, potentially revealing novel reproductive health and disease treatment avenues.

## Ethical statement

Written consent forms were received from all donors. The fetal gonad specimens were collected from terminated pregnancies, in compliance with the approval from the Ethical Committee of Nanjing Maternity and Child Health Care Hospital, China [Approval No. (2017)68]. Additionally, the use of human samples in our experiments was approved by the Ethical Committee of Nanjing Medical University, China [Approval No. (2017)582]. Exclusion criteria for the fetal gonad samples included any signs of deterioration or congestion, as well as the presence of genetic abnormalities or infectious diseases, such as hepatitis B, acquired immune deficiency syndrome (AIDS), and syphilis.

## Supplementary Material

qzaf015_Supplementary_Data

## Data Availability

The scRNA-seq data of testes obtained from donors with gestational ages of 22 and 25 weeks have been deposited in the Genome Sequence Archive [59] at the National Genomics Data Center (NGDC), China National Center for Bioinformation (CNCB) (GSA: HRA008838), and are publicly accessible at https://ngdc.cncb.ac.cn/gsa. The HumanTestisDB database is freely available for non-commercial use at https://shalab.njmu.edu.cn/humantestisdb. It has also been submitted to Database Commons [60] at the NGDC, CNCB, which is publicly accessible at https://ngdc.cncb.ac.cn/databasecommons/database/id/9721. Users are able to access any data and visualizations without registration or login.

## CRediT author statement

**Mengjie Wang:** Investigation, Data curation, Formal analysis, Methodology, Visualization, Writing – original draft. **Laihua Li:** Resources, Writing – original draft. **Qing Cheng:** Resources. **Hao Zhang:** Conceptualization, Methodology. **Zhaode Liu:** Resources. **Yiqiang Cui:** Resources. **Jiahao Sha:** Conceptualization, Funding acquisition, Project administration, Resources, Supervision, Writing – review & editing. **Yan Yuan:** Conceptualization, Funding acquisition, Project administration, Supervision, Writing – review & editing. All authors have read and approved the final manuscript.
