# Supplementary material for: HumanTestisDB: A Comprehensive Atlas of Testicular Transcriptomes and Cellular Interactions
Source: Genomics Proteomics Bioinformatics. 2025 Mar 5;23(1):qzaf015. doi: 10.1093/gpbjnl/qzaf015 (PMC12221866; doi:10.1093/gpbjnl/qzaf015)
Supplement: qzaf015_Supplementary_Data [file qzaf015_supplementary_data.zip › Supplementary material captions.docx]

**Supplementary material**

**Figure S1 Quality control of single-cell RNA-seq datasets**

**A.** Bar plot displaying the number of cells passing and failing quality control for each dataset. **B.** Violin plots depicting single-cell RNA-seq quality metrics: total molecules detected per cell (nCount_RNA), gene count per cell (nFeature_RNA), mitochondrial gene percentage (percent.mito), and ribosomal gene percentage (percent.ribo).

**Figure S2 Expression patterns of additional representative markers related to cell types in Figure 2A**

**Figure S3 Gene expression difference across cell types in Figure 2A**

Heatmap showing differentially expressed genes, with regions corresponding to transcription factors, cell surface proteins, or enzymes marked in green, and enriched biological processes for each gene cluster displayed.

**Figure S4 Characterization of the process from PGC to early Spermatocyte**

**A.** and **B.** UMAP plot of the “GermCells_part1” cell set in Figure 3A, colored by age (A) or cell type (B). Line thickness between cell types in (B) indicates connection strength. **C.** Expression patterns of classical markers for cell types in (B). **D.** Cell annotation from different literature projected on the UMAP plot of the “GermCells_part1” cell set. **E.** Heatmap showing differentially expressed genes of undifferentiated spermatogonia in (B), with transcription factors, cell surface proteins, or enzymes highlighted in green, and enriched GO biological processes and Reactome pathways for each gene cluster displayed.

**Figure S5 Dynamics during the differentiation from pre-Leptotene to early Spermatid**

**A.** UMAP plot of the “GermCells_part2” cell set in Figure 3A, colored according to cell cycle phase. **B.** Expression patterns of classical markers for cell types in the “GermCells_part2” cell set. **C.** Violin plots showing nCount_RNA, nFeature_RNA, percent.mito, and percent.ribo of each cell type in the “GermCells_part2” cell set.

**Figure S6 Gene expression dynamics throughout spermiogenesis**

**A.** Expression profiles of classical markers for cell types in the “GermCells_part3” cell set. **B.** Violin plots showing nCount_RNA and nFeature_RNA of each cell type in the “GermCells_part3” cell set. **C.** Heatmap illustrating genes dynamically expressed along the developmental trajectory from RS-1 to Sperm, with regions corresponding to transcription factors, cell surface proteins, or enzymes marked in green. The gene types included in each gene cluster are also displayed.

**Figure S7 Gene Expression insights and dynamics in somatic cells**

**A.** Heatmap showing differentially expressed genes for each cell type in Figure 4A, with transcription factors, cell surface proteins, or enzymes highlighted in green. Enriched biological processes for each gene cluster are also displayed. **B.** and **C.** Heatmap illustrating genes dynamically expressed along developmental trajectories, with transcription factors, cell surface proteins, or enzymes highlighted in green. Enriched biological processes for each gene cluster are also displayed. From Interstitial precursor-2 to Interstitial precursor-3 and from Interstitial precursor-2 to Fetal Leydig cell (B). From Leydig-PTM precursor to Leydig cell and from Leydig-PTM precursor to PTM cell (C).

**Figure S8 UMAP Partitioning based on age group, with cells colored by cell type**

**Figure S9 Statistical plot showing the distribution of each cell type of the “AllCells” cell set in each age group**

The size of the dots is proportional to the percentage of cells.

**Table S1 Detailed Information on data utilized by HumanTestisDB**

**Table S2 Differentially expressed genes of undifferentiated Spermatogonia in Figure S4B**

**Table S3 Key genes linked to functional biological events in spermiogenesis, as identified in the dynamic gene expressions of Figure S6C**
