# Supplementary figures and images for: HumanTestisDB: A Comprehensive Atlas of Testicular Transcriptomes and Cellular Interactions
Source: Genomics Proteomics Bioinformatics. 2025 Mar 5;23(1):qzaf015. doi: 10.1093/gpbjnl/qzaf015 (PMC12221866; doi:10.1093/gpbjnl/qzaf015)

A

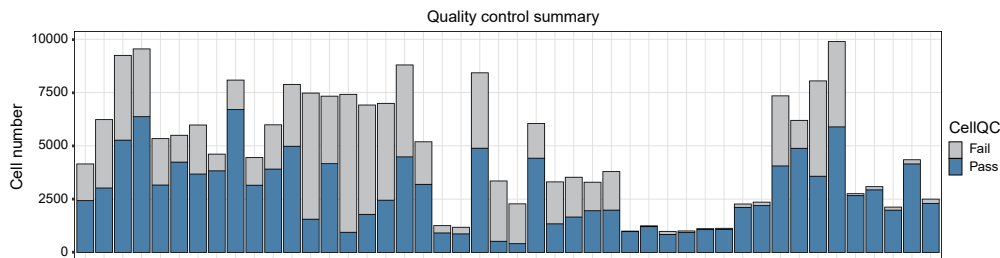

B

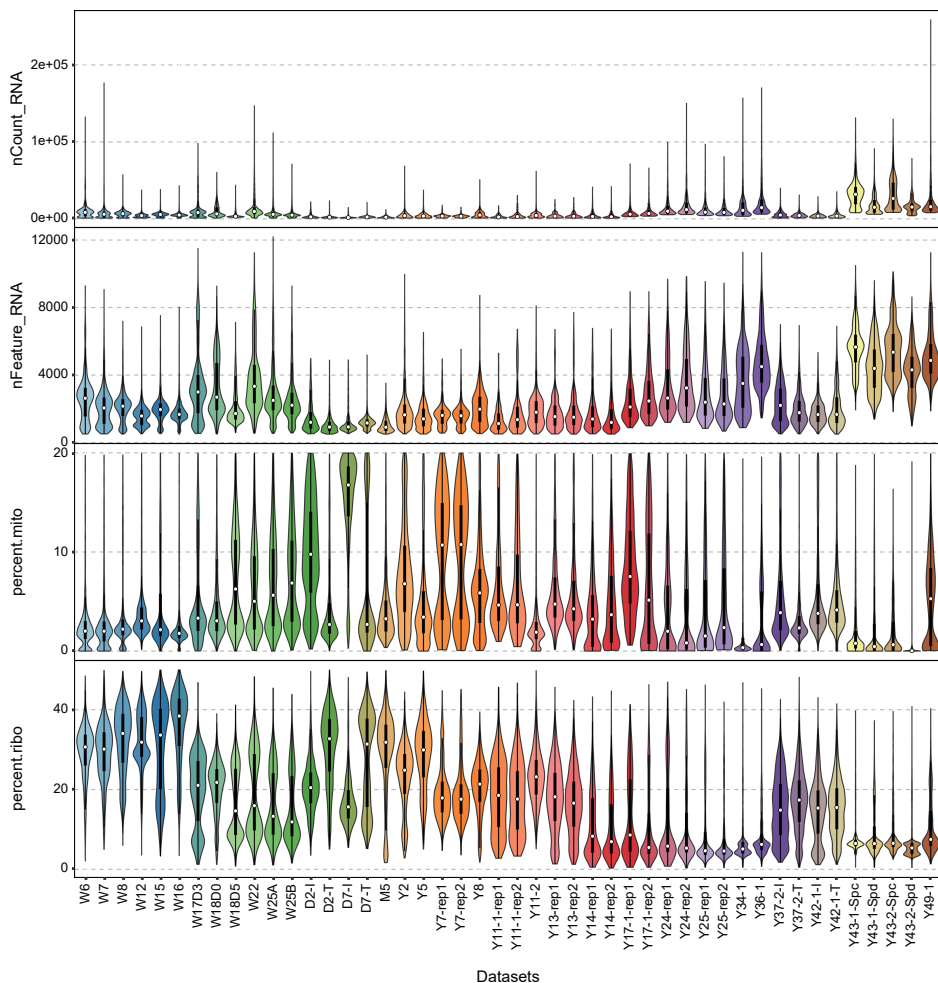

Supplement: qzaf015_Supplementary_Data [file qzaf015_supplementary_data.zip › FigureS1.pdf]

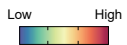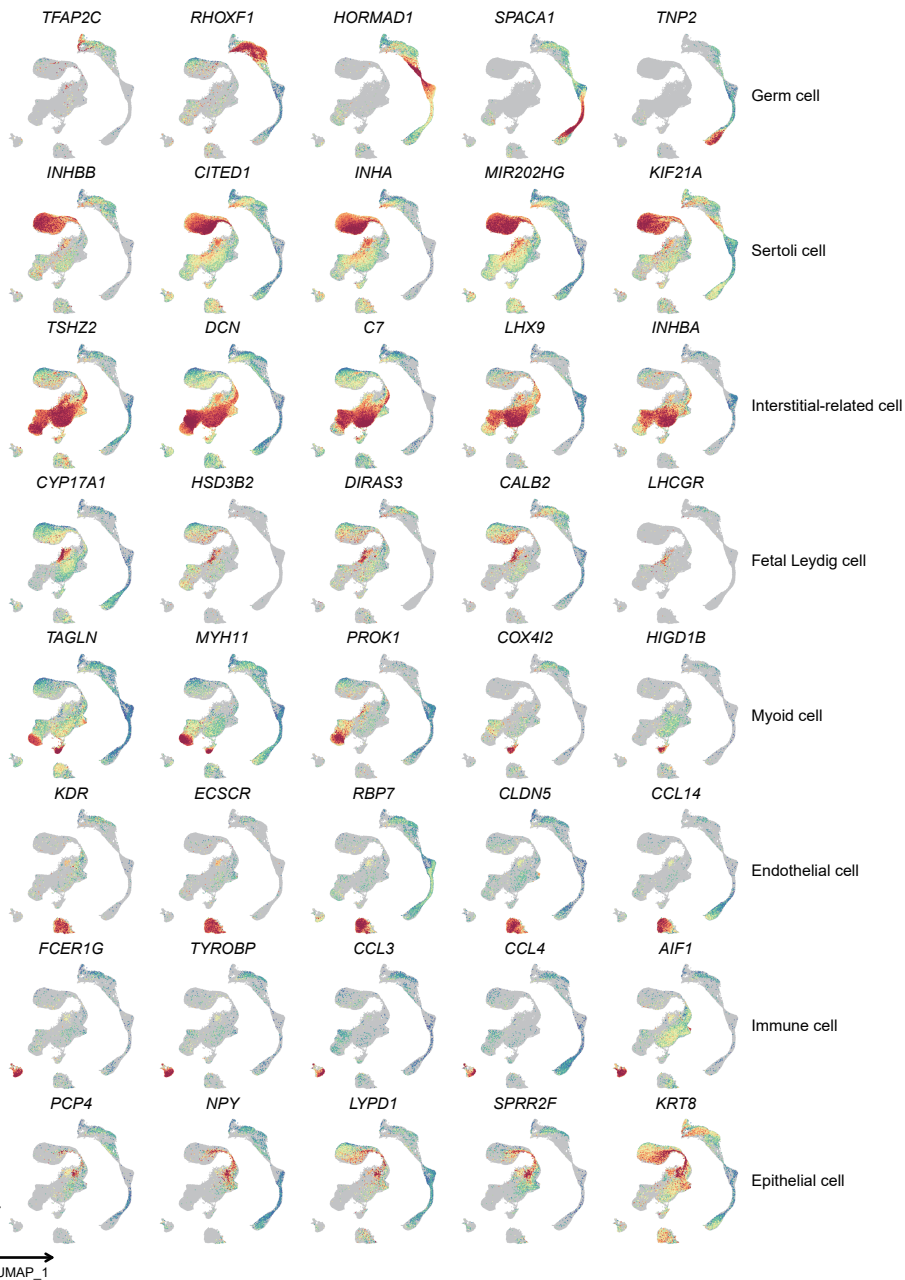

Supplement: qzaf015_Supplementary_Data [file qzaf015_supplementary_data.zip › FigureS2.pdf]

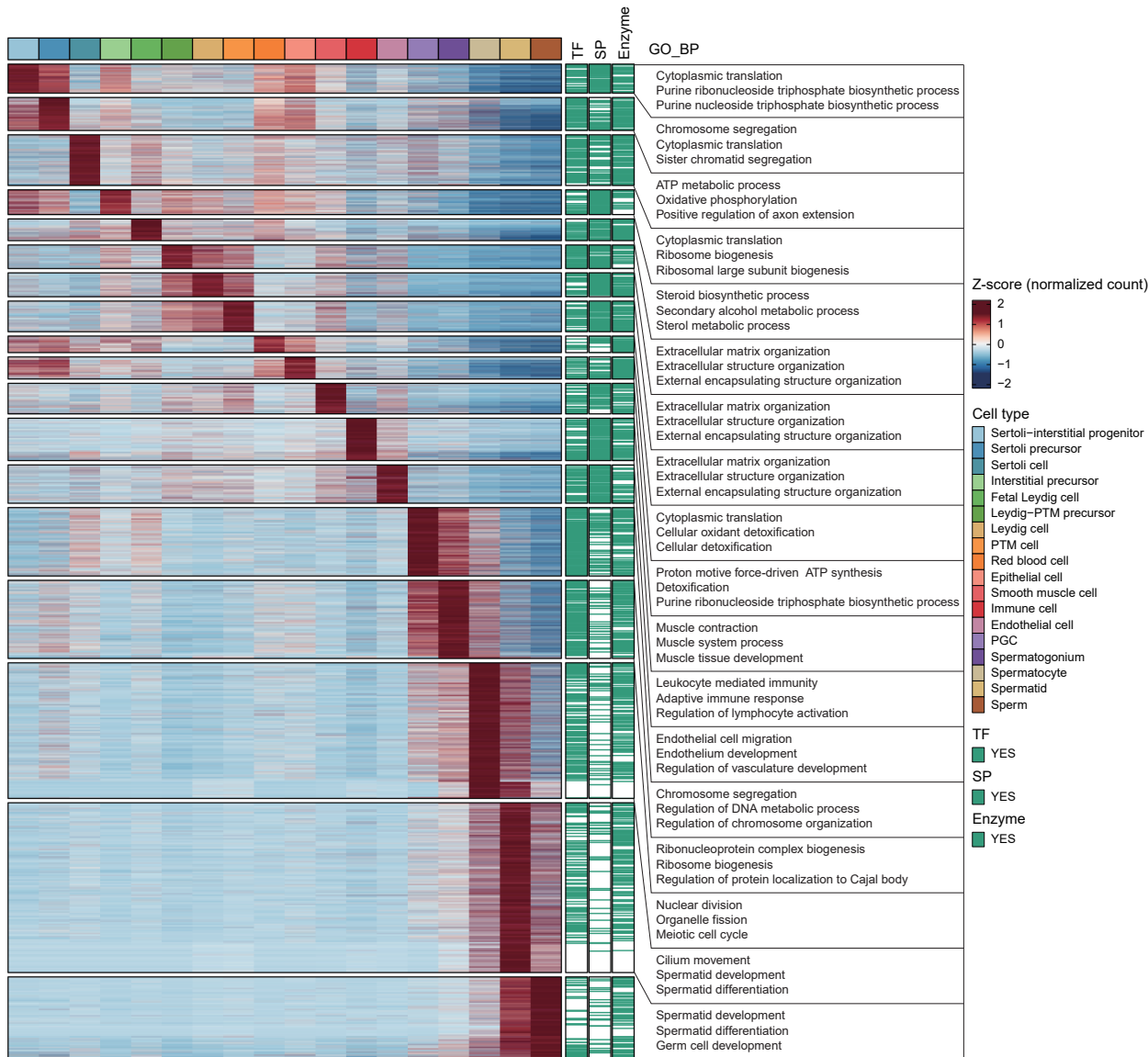

Supplement: qzaf015_Supplementary_Data [file qzaf015_supplementary_data.zip › FigureS3.pdf]

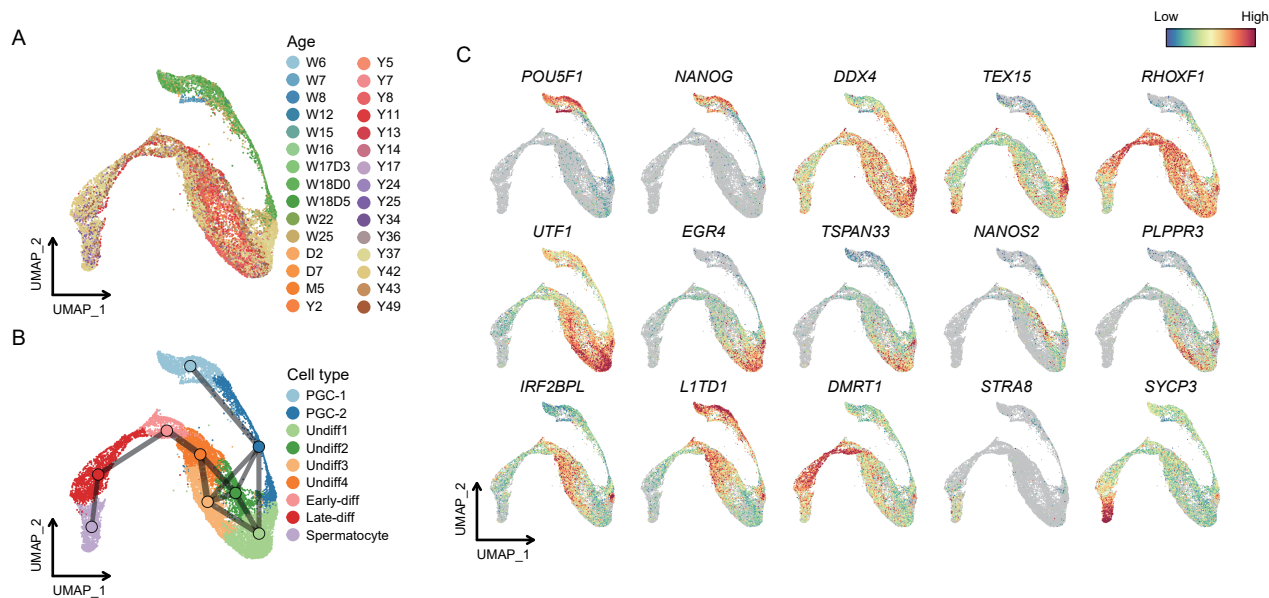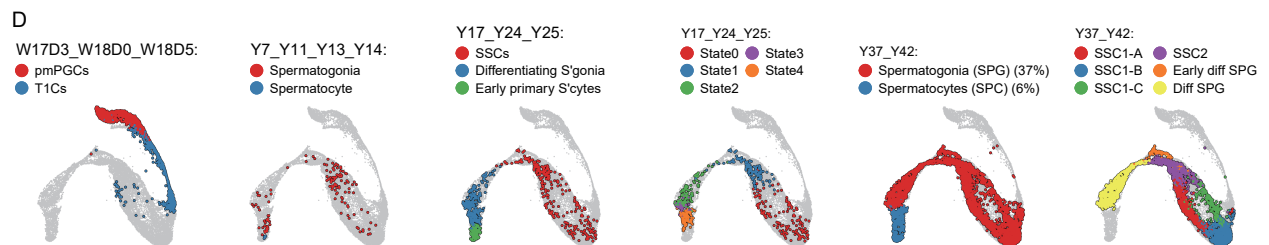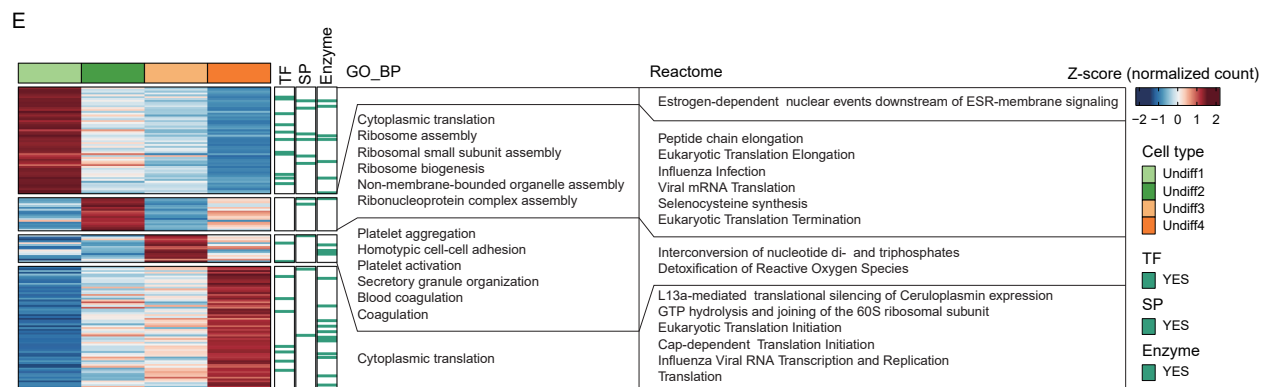

Supplement: qzaf015_Supplementary_Data [file qzaf015_supplementary_data.zip › FigureS4.pdf]

A

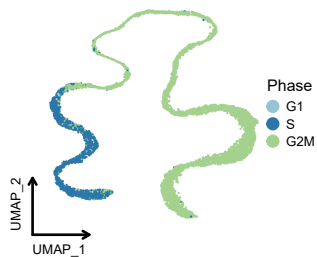

B

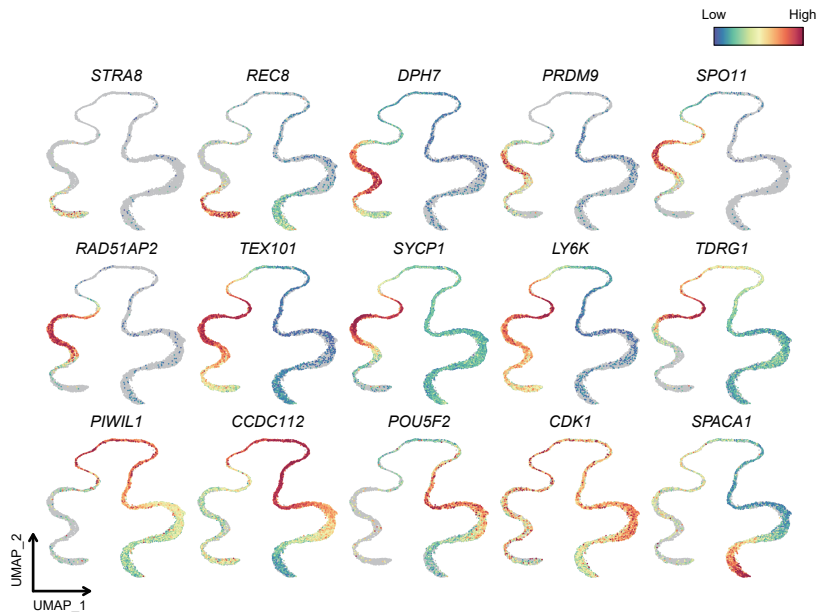

C

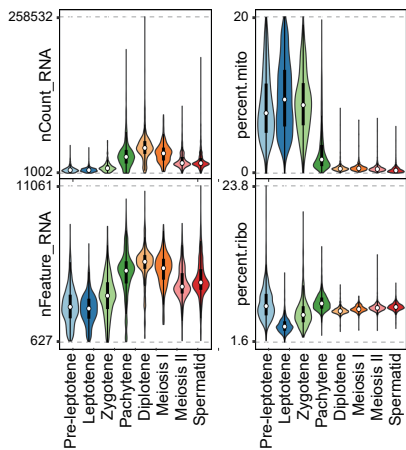

Supplement: qzaf015_Supplementary_Data [file qzaf015_supplementary_data.zip › FigureS5.pdf]

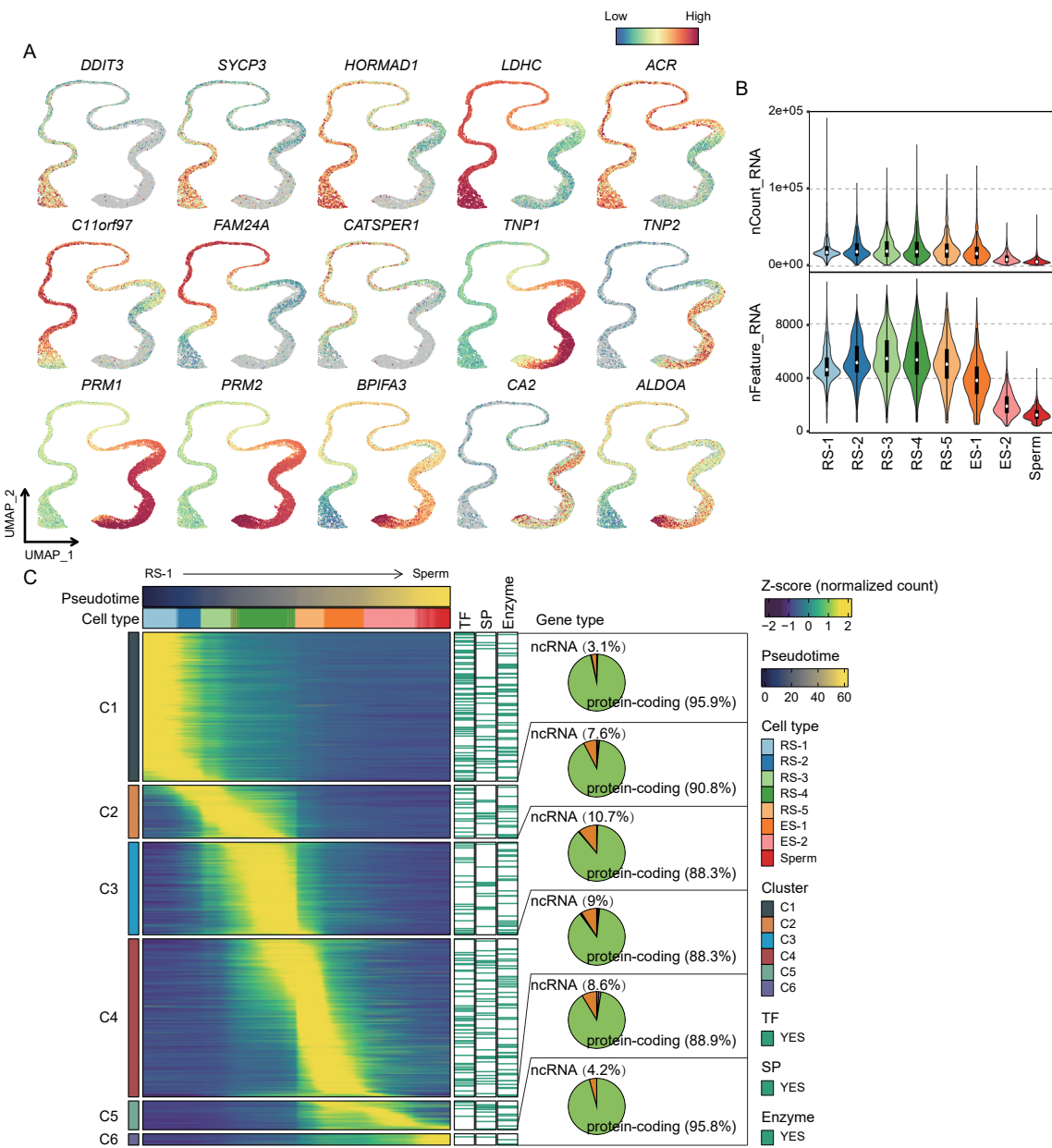

Supplement: qzaf015_Supplementary_Data [file qzaf015_supplementary_data.zip › FigureS6.pdf]

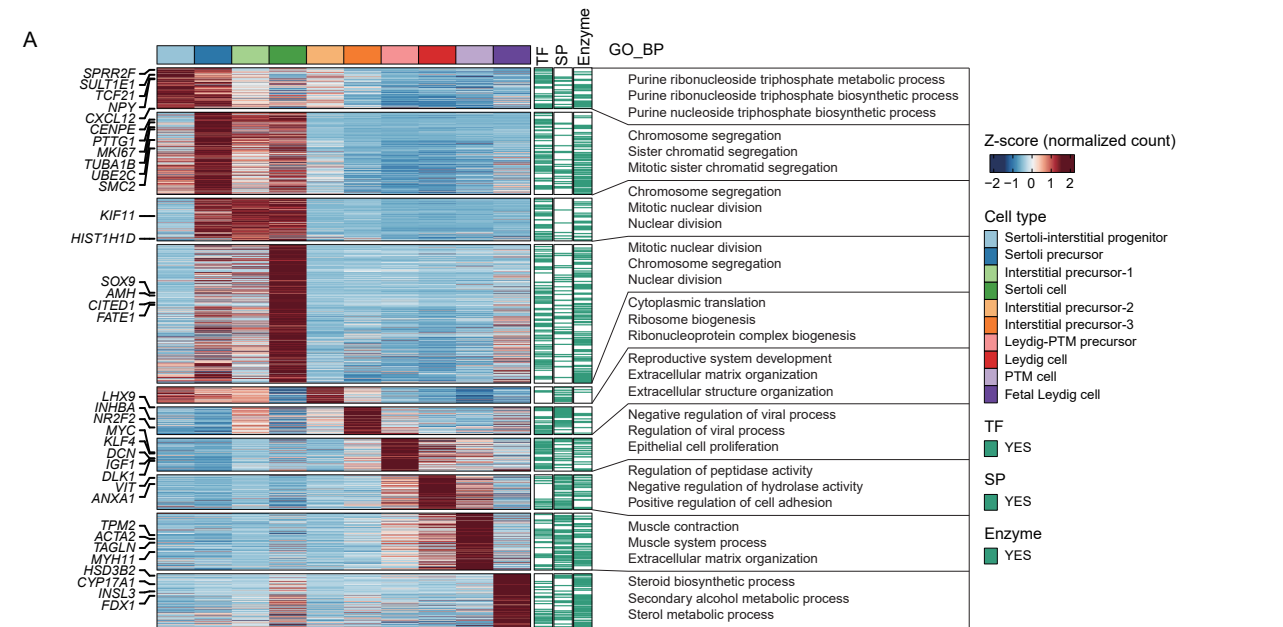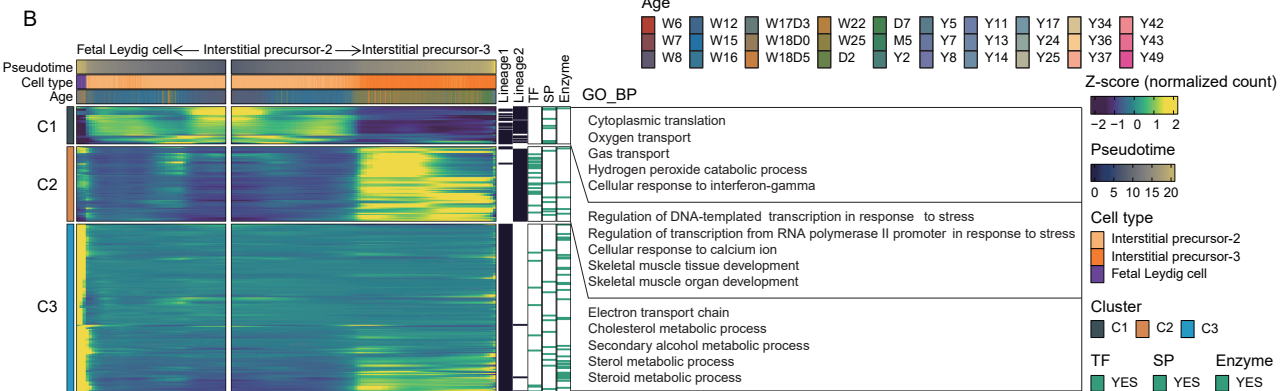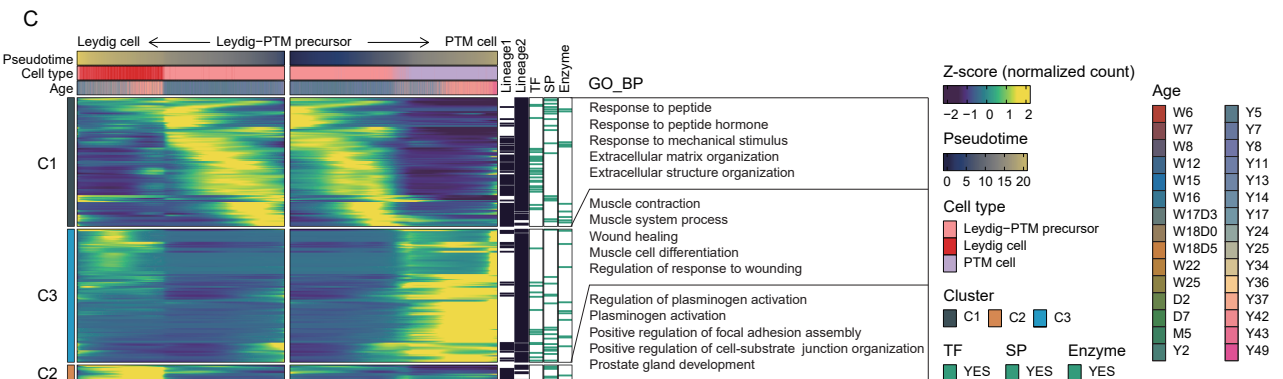

Supplement: qzaf015_Supplementary_Data [file qzaf015_supplementary_data.zip › FigureS7.pdf]

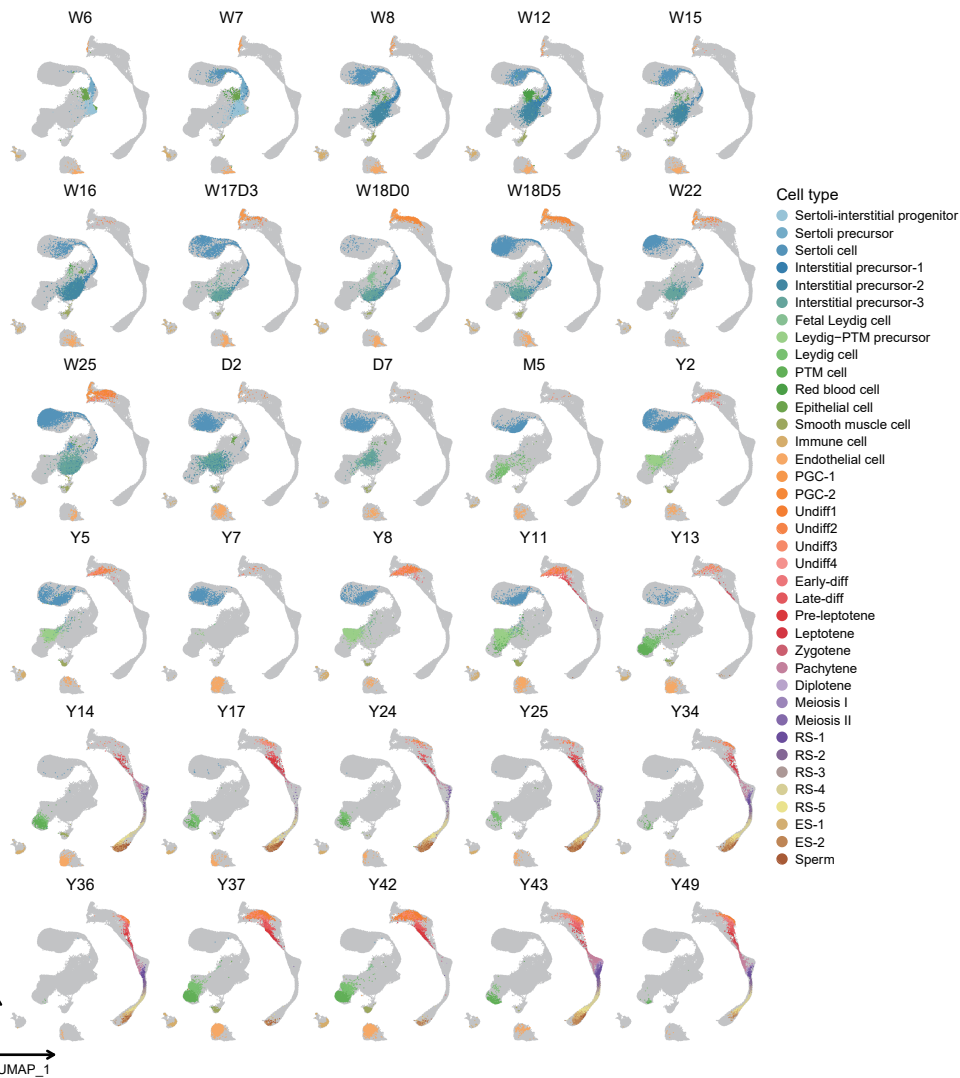

Supplement: qzaf015_Supplementary_Data [file qzaf015_supplementary_data.zip › FigureS8.pdf]

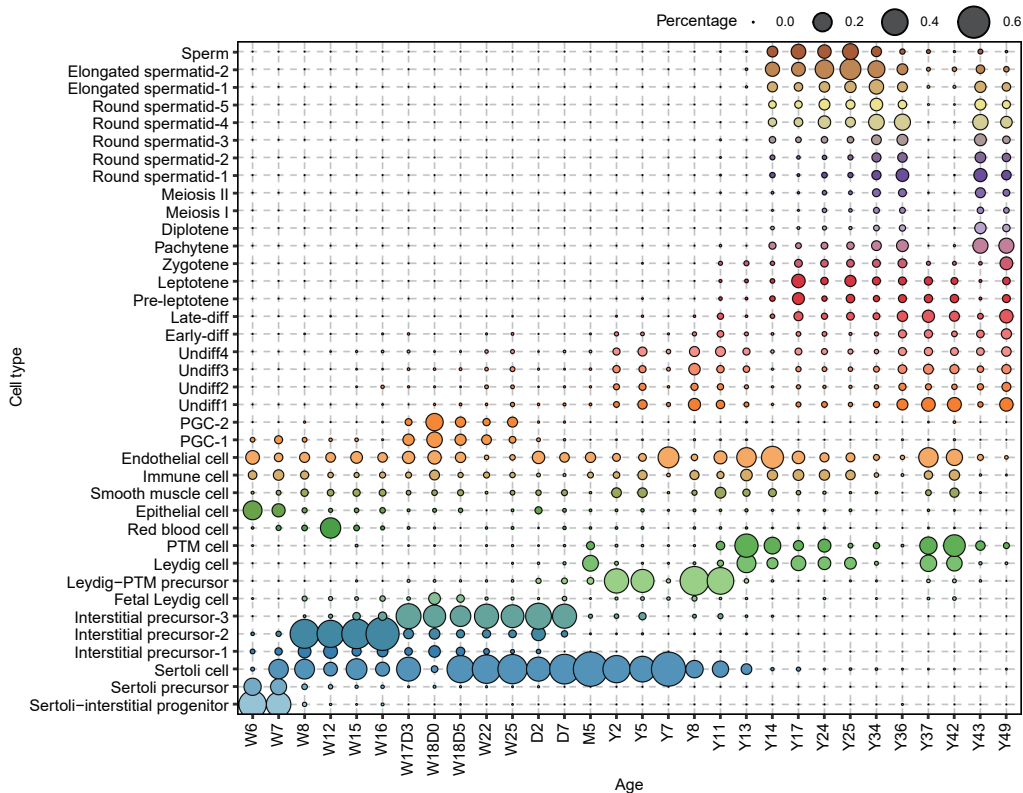

Supplement: qzaf015_Supplementary_Data [file qzaf015_supplementary_data.zip › FigureS9.pdf]
